# Intra- and Inter-Individual Variance of Gene Expression in Clinical Studies

**DOI:** 10.1371/journal.pone.0038650

**Published:** 2012-06-18

**Authors:** Wei-Chung Cheng, Wun-Yi Shu, Chia-Yang Li, Min-Lung Tsai, Cheng-Wei Chang, Chaang-Ray Chen, Hung-Tsu Cheng, Tzu-Hao Wang, Ian C. Hsu

**Affiliations:** 1 Division of Pediatric Neurosurgery, Neurological Institute, Taipei Veterans General Hospital, Taipei, Taiwan; 2 Institute of Statistics, National Tsing Hua University, Hsinchu, Taiwan; 3 The Division of Infectious Diseases, National Health Research Institutes, Miaoli, Taiwan; 4 Institute of Athletics, National Taiwan Sport University, Taichung, Taiwan; 5 Department of Biomedical Engineering and Environmental Sciences, National Tsing Hua University, Hsinchu, Taiwan; 6 Institute of Nanoengineering and Microsystem, National Tsing Hua University Hsinchu, Taiwan; 7 Genomic Medicine Research Core Laboratory, Chang Gung Memorial Hospital, Taoyuan, Taiwan; 8 Department of Obstetrics and Gynecology, Lin-Kou Medical Center, Chang Gung Memorial Hospital and Chang Gung University, Taoyuan, Taiwan; Georgia Institute of Technology, United States of America

## Abstract

**Background:**

Variance in microarray studies has been widely discussed as a critical topic on the identification of differentially expressed genes; however, few studies have addressed the influence of estimating variance.

**Methodology/Principal Findings:**

To break intra- and inter-individual variance in clinical studies down to three levels–technical, anatomic, and individual–we designed experiments and algorithms to investigate three forms of variances. As a case study, a group of “inter-individual variable genes” were identified to exemplify the influence of underestimated variance on the statistical and biological aspects in identification of differentially expressed genes. Our results showed that inadequate estimation of variance inevitably led to the inclusion of non-statistically significant genes into those listed as significant, thereby interfering with the correct prediction of biological functions. Applying a higher cutoff value of fold changes in the selection of significant genes reduces/eliminates the effects of underestimated variance.

**Conclusions/Significance:**

Our data demonstrated that correct variance evaluation is critical in selecting significant genes. If the degree of variance is underestimated, “noisy” genes are falsely identified as differentially expressed genes. These genes are the noise associated with biological interpretation, reducing the biological significance of the gene set. Our results also indicate that applying a higher number of fold change as the selection criteria reduces/eliminates the differences between distinct estimations of variance.

## Introduction

Over the last decade, microarray studies have had a profound impact on transcriptomic research. One particularly important clinical application of microarray technology is the identification of differentially expressed genes, which may serve as biomarkers for the diagnosis and prognostic prediction of tumors or other complex diseases [1]–[3]. Despite many successful results, some studies have revealed that gene lists derived from similar studies are highly inconsistent [4]–[6]. Numerous investigations have been conducted to evaluate the influence of multiple factors, such as batch effects [7], dye effects [8], different platforms [9]–[13], various experiment designs [14]–[16], and statistical approaches [17], [18], regarding microarray results. However, few studies have explored the influence of different sources of variation on the identification of differentially expressed genes from microarray analysis.

Researchers have identified two major sources of variance in microarray studies: technical variance and biological variance [19]. All forms of variations influenced by experimental artifacts, such as the quality of RNA, batch effects, and experimental parameters, belong to technical variance. A well-conceived experimental design and execution as well as rigorous statistical analysis can reduce the effects of technical variation. Studies have demonstrated that loop designs are more efficient than reference designs in two color microarrays [14], [20], and many statistical methods can be used to increase the robustness of microarray data analysis [21], [22]. Several studies have concluded that the reproducibility of microarrays could be improved using standardized protocols and carefully designed and controlled experiments [12], [13], [23].

Biological variance is attributed to specimens, rather than procedures, and can be traced to several sources. Anatomic variance is caused by the heterogeneous distribution of cell types within a tissue specimen collected from a single individual [24]. Individual variance is a result of various genotypes and physiological states. For variation in genotypes, copy number variations (CNVs) [25], [26] and allele variations [27], [28] have been shown to influence gene expression levels. Physiological status such as environment factors, disease state, and other variables influence gene expression. Many researchers have reported biological variance in human blood [29], [30], lung [31], placenta [32], retina [33], and other tissues [34]–[37]. In addition, variations in gene expression have been identified among individuals as well as populations [38]–[40] and species [19], [40], [41]. However, the effects of applying different levels of variances have not been well addressed.

In this study, we used the normal human placenta as a model to evaluate technical, anatomic, and individual variance. Each of these types of variation should be considered in clinical studies. The “inter-individual variable gene” was used as an example to evaluate the influence of estimating variance on microarray results. We profiled three levels of variance in human clinical studies and addressed the importance of estimating variance on the statistical and biological aspect for microarray studies. Our data demonstrated that correct variance evaluation is critical in selecting significant genes.

## Materials and Methods

### Specimen Collection and Processing

**Figure 1:**
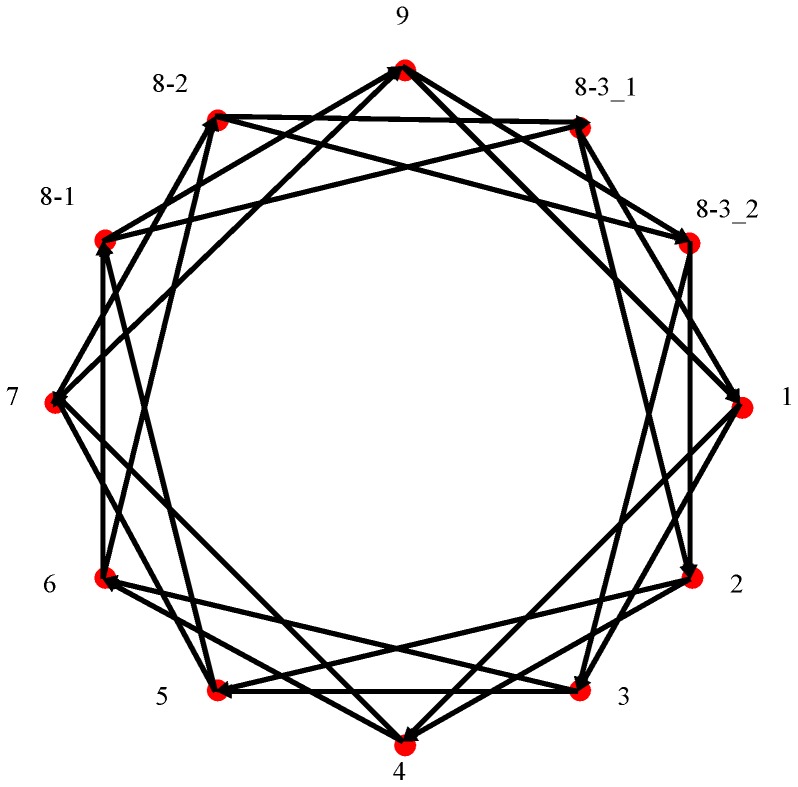
Microarray experimental design. Three kinds of samples were employed in this study. Individual variance was evaluated using the first sample group (G1), comprising Samples 1 to 9 of nine individuals. The second sample group (G2) was used to evaluate anatomic variance. It contained Samples 8–1, 8–2, and 8–3, taken from three different sections of placenta from the same individual. The third sample group (G3) consists of two technical replicates, Samples 8–3_1 and 8–3_2, using an identical RNA pool for microarray hybridization to evaluate technical variance. The expression of Sample 8–3 could be estimated by the mean expression of Samples 8–3_1 and 8–3_2. The mean expression of Samples 8–1, 8–2, and 8–3 represented the expression of Sample 8.

Eleven normal placental tissues were obtained from 9 healthy individuals who underwent cesarean section without labor pain [42]. This study was approved by the Institutional Review Board of Chang Gung Memorial Hospital (IRB#96-0630B). Inclusion criteria were healthy normotensive term pregnancies with appropriate-for-gestational-age fetuses, who displayed no abnormality on routine ultrasound scans. Exclusion criteria for this study were fetal chromosomal abnormalities, pre- and postnatal malformations or phenotypic anomalies, maternal smoking, maternal obesity, and maternal diseases, such as autoimmune diseases, thrombophilic conditions, and diabetes [43]. The clinical information is summarized in Table 1. Placental specimens were obtained from the same region of the placenta (5 cm away from the site of cord insertion) immediately after delivery. The approximate 2.5-cm thickness of the placental cross section was divided into three equal parts: maternal (includes thin basal plate), middle, and fetal (includes the chorionic plate) [32]. We analyzed the middle part of the placental tissues in all of our placental studies [42], [44]. The tissues were snap frozen in liquid nitrogen and stored at −80°C. The first sample group (G1) comprised samples 1 to 9 of 9 individuals. The second sample group (G2) contained 8–1, 8–2, and 8–3, which were 3 different placental tissues taken from the same individual. The third sample group (G3) consisted of 2 technical replicates, 8–3_1 and 8–3_2, using the identical RNA pool (Figure 1).

**Table 1:** Clinical information of pregnancy outcomes (n = 9).

Clinical parameter	Mean ± SD*	Range of this group	Reference range
Maternal age (y)	32.6±3.7	25 ∼ 36	NA
Gravida#	2.6±1.1	1 ∼ 4	NA
Para&	1.2±0.8	0 ∼ 2	NA
Maternal Hemoglobin (g/dL)	10.8±1.8	8.6 ∼ 13.5	12 ∼ 16
Mean cell volume of RBC (fL)	83±7.6	72 ∼ 92	80 ∼ 100
Systolic blood pressure (mmHg)	117.1±11.4	102 ∼ 136	90 ∼ 140
Diastolic blood pressure (mmHg)	62.4±10.3	50 ∼ 78	50 ∼ 90
Gestational age (week)	38.3±0.9	37 ∼ 39	38 ∼ 40
Neonate body weight (g)	3133±345	2520 ∼ 3580	2430 ∼ 3900
Apgar score% (1 min)	9.0±0.5	8 ∼ 10	>7
Apgar score% (5 min)	9.9±0.3	9 ∼ 10	>7

# indicates the number of times the mother has been pregnant, regardless of whether these pregnancies were carried to term. A current pregnancy, if any, is included in this count.

& indicates the number of viable (>20 wks) births. Pregnancies consisting of multiples, such as twins or triplets, count as ONE birth for the purpose of this notation.

% is a simple and repeatable method to quickly and summarily assess the health of newborn children immediately after birth.

* is standard variation.

### RNA Extraction and Microarray Hybridization

Total RNA was isolated as previously reported [45]. Because the purpose of this study was to analyze variance of gene expression that may be commonly encountered at the tissue level, we did not isolate individual cell types from whole tissues. During RNA extraction, 1 ml of Trizol reagent (Life Technologies, Rockville, MD) was added to every 50–100 mg of pulverized frozen placental tissue. Total RNA was isolated using the Trizol reagent (Life Technologies, Rockville, MD). Total RNA was quantified by UV absorption at 260 nm, and RNA quality was examined using the Agilent 2100 bioanalyzer (Agilent technologies, USA). cDNA labeling was conducted using a 3 DNA Array 50™ kit (Genisphere, Hatfield, PA), according to the manufacturer’s protocols. In brief, 20-µg total RNA was used to perform reverse transcription reaction with SuperScript II RNase H- reverse transcriptase and specific primers (Invitrogen life technologies, USA). All synthesized tagged cDNA targets were then purified using the Microcon YM-30 column (Millipore, USA). The purified targets and fluorescent 3 DNA reagents were hybridized to the arrays in succession. Arrays were sealed in a homemade hybridization chamber that adapted the design provided in M-Guide (Patrick O. Brown laboratory, Stanford University, USA). Hybridization was performed at 65°C in a water bath for 16 h, and arrays were washed according to the manufacturer’s protocol (http://www.genisphere.com/pdf/array50v2_10_19_04.pdf). Subsequently, arrays were scanned with GenePix 4100A (Axon Instruments, USA) and images were acquired using GenePix Pro 5.0 software (Axon Instruments, USA).

### Production of Microarrays

We originally ordered 9600 human cDNA clones of the IMAGE library from Incyte Genomics (Palo Alto, Calif, USA) and allowed sequencing at that location. Only 7334 clones passed sequence verification by Incyte Genomics and were shipped to us. Therefore, every clone of this 7334-clone cDNA library had an IMAGE ID, DNA sequences, vector names, and information for PCR primers [45] **.** All clones were further amplified by PCR and purified by isopropanol precipitation in 96-well plates. The purified DNAs were resuspended in 3×SSC for spotting. A single microarray slide (CMT-GAPsII, Corning Inc., USA) contains 7334 human cDNA probes in quadruplicate, 10 spike-in genes (SpotReportTM-10 Array Validation System, Stratagene, USA), and one housekeeping gene, β-actin, in 96 replicates. Each array had 32,448 spots. The arrays were post-processed as recommended in the Corning UltraGAPS Coated Slides Instruction Manual. Microarray slides were produced in a well-controlled environment (28±2°C and 48±1% humidity) and stored under desiccation until use. The array system was assembled according to M-Guide (Patrick O. Brown laboratory, Stanford University, USA) and controlled using ArrayMaker, version 2.5.1 (Joseph DeRisi laboratory, UCSA, USA) [46]. A rigorous system commissioning was performed to guarantee the quality of the printed arrays. Before hybridization, the slides were preprocessed according to the instruction manual for the Corning UltraGAPS Coated Slides, including rehydration, snap-dry, UV-crosslinking, baking, and surface blocking. DNAs were UV-crosslinked with 300 mJ/cm2 using the Stratalinker 2400 UV Crosslinker (Stratagene, USA).

### Microarray Data Analysis

The logarithm of the ratios for all valid spots on each array was normalized by locally weighted linear regression (LOWESS). Descriptions of Microarray Data Preprocessing can be found in our previous studies [47]. The normalized log ratios were then processed gene-by-gene using a log linear model [47], [48]. This model describes the normalized log ratio as follows:
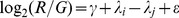
where γ represents the relative labeling efficiency between dyes, λ_i_ is log2 (expression of sample i/mean expression of all samples) for one specific cDNA clone, with 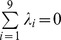, and ε is the random error with mean 0 and variance σ^2^. σ represents the estimated variance for one specific cDNA clone. For each clone, λ_i_ and σ are estimated from the observed data by using the least squares method as 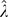 and 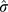. When the data had been processed using the log linear model, 5501 genes could be calculated in the model without singularity. 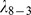 is estimated by 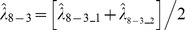. 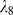 is estimated by 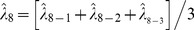. A further description of the statistical model can be found in Methods S1. We had developed a Web tool for loop-design microarray data analysis [49]. All of the front-end analyses of our microarray data were conducted using this public available Web tool. The microarray data of this work are MIAME compliant and have been deposited in the GEO of NCBI (accession number: GSE27646).

### Differential Expression and Averaged Fold Change

**Figure 2:**
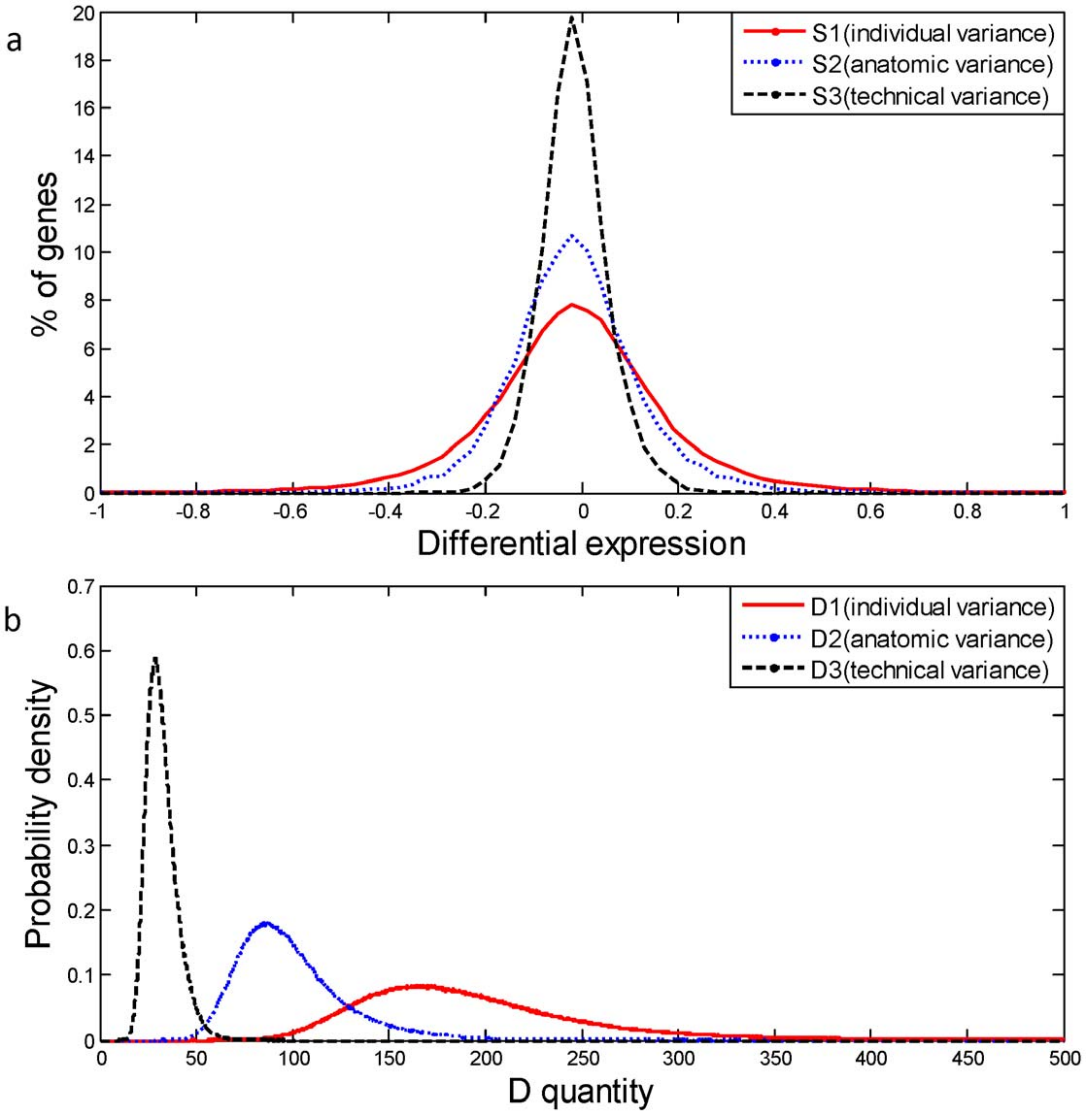
Profiles of the three kinds of variance. (a) The distribution of the differential expression for the three forms of variance. The differential expression for the three forms of variance was estimated by S1: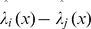, S2:
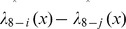
, and S3: 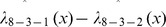 for any possible pair of i and j, respectively. (b) D1, D2, and D3 are the probability density distributions of D quantity using permutation method using the data series S1, S2, and S3 when considering individual, anatomic, and technical variance respectively.



Differential expression is log2 (fold change of 2 samples) for one specific cDNA clone and is denoted as 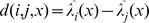, where *x* is the index denoting clones and *i,j* denoting samples. Differential expression profiles in Figure 2a are the histograms of data set S1: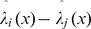, S2:
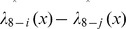
, and S3: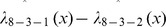, which are the set of all 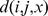 when *x* runs over all clones and (*i,j*) runs over all possible pairs in G1, G2, and G3, respectively. For S1, *i* and *j* range from 1 to 9. For S2, i and j range from 8–1 to 8–3. For S3, i and j are 8–3_1 and 8–3_2, respectively. Moreover, averaged fold change is estimated by.

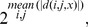
where 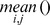 denotes the mean over absolute expression differences of all possible sample pairs (*i,j*) for clone *x*. It is the indicator of fold change for individual variance.

### Statistical Test

We designed a test statistic,



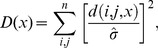
to describe the variation of gene expression between samples. The summation runs on every dual-color microarray experiment (represented by an arrow in Figure 1), where x is the *xth* clone, *i* is for the sample represented by the tail of the arrow, *j* is for the sample represented by the head of the arrow, and n is the number of sample pair i,j. We used the sampling permutation method to describe the D quantity when considering three levels of variance (Methods S1). D1, D2, and D3 are the results of 10 million times the sampling permutation of 
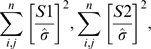
 *and* 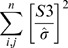, for taking n data from S1, S2, and S3 at one time. The corresponding *p* values of the D quantity are determined using the smoothed curve of the probability density in Figure 2b. The criterion of the *p* value for the statistical test in this study is a false discovery rate (FDR) of 5%.

### Functional Enrichment Analysis

Gene Ontology (GO)-based functional enrichment analysis is used to measure gene enrichment in annotation terms for the inter-individual variable genes. The significance score in Table 1 is –log (EASE Score), where the EASE Score is a modified Fisher exact *p* value [50] obtained by DAVID. The GO terms passed the criteria, EASE Score <0.1, and at least 2 genes in each GO term are considered for further comparison. Only 11 mutual GO terms exist for all selection criteria, and these are shown in Table 2.

**Table 2:** Significant score of Gene Ontology terms for the significant gene sets determined by distinct significant criteria.

Gene Ontology terms	1.2 fold§		1.3 fold§	
	Tech¥	Ana#	Tech¥	Ana#
GO:0005576∼extracellular region	8.7	9.3	4.9	4.6
GO:0005615∼extracellular space	7.8	8.5	4.8	5.0
GO:0006952∼defense response	7.4	8.1	5.3	5.5
GO:0044421∼extracellular region part	6.4	7.2	4.2	4.4
GO:0007565∼female pregnancy	2.8	3.0	2.2	2.3
GO:0009617∼response to bacterium	2.4	2.6	1.6	1.6
GO:0050832∼defense response to fungus	2.4	2.4	3.1	3.1
GO:0031640∼killing of cells of another organism	2.3	2.4	3.0	3.0
GO:0001906∼cell killing	1.8	1.9	2.5	2.6
GO:0009620∼response to fungus	1.8	1.9	2.5	2.6
GO:0042445∼hormone metabolic process	1.3	1.4	2.3	2.3

The number in the table is the significant sore for GO terms. The significant score is –log (EASE Score) where EASE Score is a modified Fisher Exact P Value obtained by DAVID.

§ The criteria of averaged fold change.

¥ The significant score is evaluated by technical variance.

# The significant score is evaluated by anatomic variance.

## Results

### Demographics of Studied Subjects

Analyzed placental tissues were collected from 9 healthy pregnant women, whose clinical information is listed in Table 1. All the pregnant women were free of hypertension, diabetes mellitus, preterm labor, and other medical diseases. All neonates were born at term and with normal body weight and healthy vital signs that were evaluated with Apgar scores at 1 min and 5 min after delivery, as used previously [42]–[44].

### The Profiles of 3 Levels of Variance

We used a loop design in a microarray analysis of normal placental tissues to investigate technical, anatomic, and individual variance in microarray data. Figure 1 is a schematic representation of the interwoven loop hybridization design performed in this study. We selected 11 normal placental tissues from 9 women with term pregnancies, who underwent Cesarean section prior to the onset of labor, to avoid variations caused by labor pain. Microarray data were obtained from 3 sample groups to estimate individual, anatomic, and technical variance. The first sample group (G1) comprised Samples 1 to 9, samples of 9 individuals. The second sample group (G2) contained Sample 8–1, 8–2, and 8–3, which were 3 different placental regions taken from the same individual. The third sample group (G3) consisted of 2 technical replicates, Sample 8–3_1 and 8–3_2, obtained from the same RNA pool. Differential expression profiles in Figure 2a are log (fold change) between samples in 3 sample groups (G1, G2, and G3) and it is the histogram of data series S1, S2, and S3, respectively. These results were presented as distributions of the fold changes of G1, G2, and G3. The results indicate a progressive narrowing of distribution curves from S1 to S3, revealing that individual difference produced a greater degree of relative variability in gene expression than that of the anatomic or technical difference.

A test statistic, D quantity, was designed to measure the variation in gene expression between samples. Figure 2b shows the probability density profiles of the D quantity, D1, D2, and D3, representing 3 levels of variability. These profiles were generated by applying permutation methods using the data series S1, S2, and S3, indicating extreme differences in the 3 levels of variance.

### Case Study: Inter-individual Variable Gene

In this study, inter-individual variable genes, of which the expression varies highly between individuals, were used to evaluate the importance of estimating variance. When defining inter-individual variable genes according to D quantity, variations in gene expression were set at a level exceeding that of anatomic variance. Therefore, when anatomic variance was considered in the significance test, Pa is the *p* value of the D quantity determined the D2 curve in Figure 2b. When anatomic variance is not considered in the experimental design, technical variance, evaluated by technical replication, is commonly used for the significance test. Pt is the *p* value of the D quantity determined by technical variance (D3 curve in Figure 2b).

**Figure 3:**
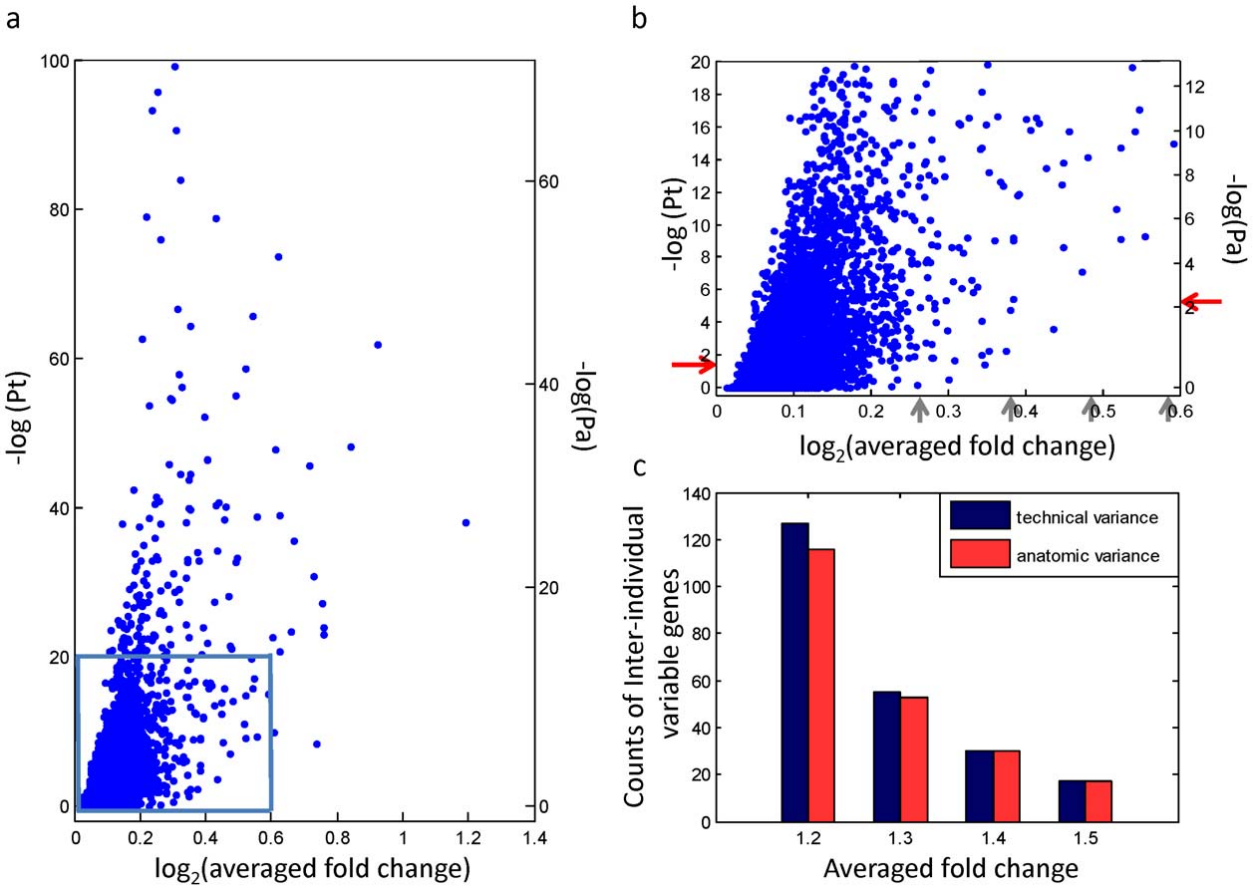
The scatter plot of averaged fold change and p values, and the selection of inter-individual variable gene. (a) The scatter plot of log_2_ (averaged fold change) and –log (p value). Pa is the p value determined by applying anatomic variance. Pt is the p value determined by applying technical variance. (b) The enlarged area of the rectangle in (a). The red arrows indicate the corresponding p value of FDR 5%. The gray arrows indicate the averaged fold change criteria: 1.2, 1.3, 1.4, and 1.5. (c) The number of inter-individual variable gene selected by the criteria of FDR 5%, evaluated by technical and anatomic variance (The red arrows in Figure 3b), and distinct averaged fold changes (The gray arrows in Figure 3b).

Figure 3a plots averaged fold change versus 2 corresponding *p* values (Pa and Pt) for each gene. When FDR 5% was set as significant, 2 groups of significant genes were obtained. The 2 corresponding cutoff *p* values are indicated by red arrows in Figure 3b. Averaged fold change was used as another criterion to select inter-individual variable genes. In this study, the 4 averaged fold changes, from 1.2 to 1.5 (the gray arrows in Figure 3b), served as further criteria for the identification of inter-individual variable genes.

We investigated sets of inter-individual variable genes generated according to distinct selection criteria (different averaged fold changes and corresponding *p* values) to evaluate the effects of differing levels of variance. Figure 3c shows the number of significant genes identified using 2 variance criteria, Pt and Pa (the red arrows in Figure 3b), with different averaged fold changes (the gray arrows in Figure 3b). When a higher averaged fold change was used, the influence of variance underestimation decreased, as shown by the number of significant genes (Figure 3c), but it paid by reducing the number of selected genes. The difference was eliminated when the cutoff value of averaged fold change was set to greater than 1.3.

To evaluate the influence of variance underestimation on biological prediction, the gene lists identified using the criteria in Figure 3c underwent functional enrichment analysis for gene ontology (GO) using DAVID bioinformatics resources 6.7 [50]. Among all significant genes listed in Figure 3c, only 11 common GO terms were identified. Table 2 shows enrichment analysis results of the 11 GO terms for the significant genes listed when applying anatomic and technical variance with the averaged fold change criteria 1.2 and 1.3. The enrichment results of averaged fold change set at 1.4 and 1.5 were not listed because 2 significant gene lists based on anatomic and technical variance were the same. A significance score was defined as -log (*p* value), where the *p* value represented the significance of each GO term, according to a modified Fisher exact test in DAVID bioinformatics resources 6.7. Hence, a higher significance score represents a higher significance for the result.

For the same GO term, the significance score for the gene set, the *p* value of which was deduced by applying anatomic variance, was usually higher than that defined by technical variance (Table 2). This suggests that the lists of significant genes based on technical variance might include “noisy” genes, which reduced the significance of the GO terms.

## Discussion

Even as simple as a single cell, its physiology are governed by various networks, each comprising multiple signaling gene products, which interact through positive and negative feedbacks, as we showed previously [51]. Complexity theory, also known as chaos theory (http://en.wikipedia.org/wiki/chaos_theory), has been developed (http://sbs-xnet.sbs.ox.ac.uk/complexity/complexity_home.asp) to better describe the emergent phenomenon of the cell. Clinical studies investigating the clinical outcomes of individuals [52] often derive results full of noise, which can be further grouped into intra- and inter-individual variance. Therefore, devising analytical approaches to dissect these confounding factors is critical.

In this study, we first collected placental tissues only from carefully selected healthy term pregnancies, avoiding any potential effects from maternal or fetal diseases. For a single organ, different regions may have distinctly specialized functions, leading to variations in gene expression [31], [32]. However, this type of variation differs between organs. The anatomic variance identified in this study was the heterogeneous distribution of cell types within a tissue specimen [53], prevalent in general clinical studies. Therefore, all tissues in this study were obtained from the same regions and same layer of the placenta to avoid biological variance among different regions of the placenta [32]. We did not isolate fetal trophoblasts from maternal endothelial cells in each placental tissue because we attempted to analyze the intra- and inter-individual variance directly from clinical tissues. To achieve this goal, we used a loop-designed method to increase the statistical power of microarray data analysis.

We used a test statistic, D quantity, in this study to describe variations in gene expression between samples. The permutation method was employed to describe the characteristics of the 3 levels of variability. Permutation analysis is frequently adopted for microarray studies [54]–[59] because distributional assumptions (e.g., normal) using microarray data are often questionable [54]. A non-parametric approach considering factors such as non-uniform distributions could exhibit the characteristics of data more appropriately. The profiles shown in Figure 2 illustrate the differences in the 3 levels of variability, demonstrating that the evaluation of the correct variance must be considered in the experimental design to define statistically significant genes.

For the selection of significant genes, the results of phase I of the MicroArray Quality Control (MAQC) project suggest that the inter-platform reproducibility of enriched KEGG pathways and GO terms was markedly increased when fold-change ranking in addition to a non-stringent *p* value cutoff were used as the selection criteria [60]. Thus, we used a non-stringent *p* value, FDR 5%, with averaged fold change as the selection criteria. However, the relationship between the stringency of fold change and biological significance remains controversial. We compared the use of 4 averaged fold changes as criteria to identify the common GO terms of all selection criteria. Pan et al. suggested that the robustness of biological conclusions derived from microarray analysis should be routinely assessed by examining the validity of the conclusions using a range of threshold parameters [61]. Hence, common GO terms are representative functions for inter-individual variable genes. In this manner, the influence of variance underestimation could be evaluated by using the significant scores of the common GO terms. The significant scores of the canonical pathways had been used to access distinct selection criteria [62].

The identification of inter-individual variable genes through different variance levels demonstrates the importance of estimating variance from the statistical and biological viewpoints. From the statistical aspect, the impact of variance underestimation includes non-statistically significant genes in the gene list (Figure 3c). From the biological aspect, significant scores of GO terms were used to evaluate the gene sets from distinct criteria. Table 2 shows a summary of biological evidence for evaluating gene sets with different significance criteria. It also shows that significant gene sets with accurate evaluation of variance provided more accurate biological interpretations. Our results also suggest that applying a higher cutoff point of fold change reduced, or even eliminated, the influence of variance underestimation. This may be a solution to overcome the difficulties associated with the identification of significant genes when the estimation of precise variance has not been considered adequately in the experimental design, although it paid by reducing the number of the final gene list.

This study demonstrated the importance of estimating variance. Different types of biological variance should be considered, depending on the objectives of a particular study. For example, when using tumor and normal tissues collected from the same individual to study the signature of a cancer [63], anatomic variance should be considered. In clinical studies seeking to identify biomarkers for cancer classification, in which the subject of the experiment is of the same race, individual variance should be considered. When experimental subjects of clinical studies include individuals from different races, inter-population variance should be considered. Different sampling contributes different levels of variance, and such factors should be considered in the experimental design and statistical model. Our results indicate that “noisy” genes are falsely identified as differentially expressed genes when the level of variance is underestimated, and applying a higher fold change as the selection criterion reduces/eliminates the differences between distinct estimations of variance.

## Supporting Information

Methods S1 **The detail description of the statistic model and sampling permutation method.**
(DOC)
