# Supplementary material for: Intra- and Inter-Individual Variance of Gene Expression in Clinical Studies
Source: PLoS One. 2012 Jun 18;7(6):e38650. doi: 10.1371/journal.pone.0038650 (PMC3377725; doi:10.1371/journal.pone.0038650)
Supplement: Methods S1 — The detail description of the statistic model and sampling permutation method. (DOC) [file pone.0038650.s001.doc]

**Statistical model**

Consider a two-color microarray experiment in which$v$samples (designated as “varieties” by some authors and called “targets” in the context of hybridization) are compared using s slides. Each spot on the slides corresponds to a particular clone. We model the data for each clone separately. With each spot on the slide in which samples $k$(labeled with Cy5) and$k'$(labeled with Cy3) are mixed and hybridized, there are associated two quantities$R$and$G$which are the normalization-corrected intensities of red (Cy5) and green (Cy3) fluorescents. We assume that the intensities are proportional to the true expression levels, denoted by$\mu_{kj}$and$\mu_{k'j}$for samples $k$and$k'$respectively, of the corresponding clone$j$. Since the data will be analyzed for each clone separately, for simplicity, we omit the suffix$j$in the following discussion and model$R$and$G$as follows:

$R=r\mu_{k}2^{\varepsilon_{r}}$, $G=g\mu_{k'}2^{\varepsilon_{g}}$,

where r and g are the proportional factors of red and green dyes respectively. $\varepsilon_{r}$and $\varepsilon_{g}$are random error terms. The logarithmic ratio

$$\begin{matrix} Y\equiv\text{log}_{2}\left(\frac{R}{G}\right)=\text{log}_{2}\left(r/g\right)+\text{log}_{2}\left(\frac{\mu_{k}}{\mu_{k'}}\right)+\left(\varepsilon_{r}-\varepsilon_{g}\right) \\ \text{ }\equiv\gamma+\text{log}_{2}\left(\frac{\mu_{k}}{\mu_{k'}}\right)+\varepsilon, \end{matrix}$$

where the parameter$\gamma\equiv\text{log}_{2}\left(r/g\right)$ represents the relative labeling efficiency between dyes. Let $\overline{\mu}=\left(\mu_{1}\mu_{2}\cdots\mu_{v}\right)^{\frac{1}{v}}$ be the geometric average of the true expression levels$\mu_{1},\mu_{2},\cdots,\mu_{v}$of the $v$ samples, then

$Y=\gamma+\text{log}_{2}\left(\frac{\mu_{k}}{\overline{\mu}}\right)-\text{log}_{2}\left(\frac{\mu_{k'}}{\overline{\mu}}\right)+\varepsilon\equiv\gamma+\lambda_{k}-\lambda_{k'}+\varepsilon$,

where parameters $\lambda_{k}\equiv\text{log}_{2}\left(\frac{\mu_{k}}{\overline{\mu}}\right)$ , $k$=1, 2, …, $v$ , represent the relative expression levels among$v$samples. It is easy to see that $\sum_{k=1}^{v} \lambda_{k}=0$. Among them, there are only$v-1$independent parameters$\lambda_{1},\lambda_{2},\cdots,\lambda_{v-1}$and$\lambda_{v}=$ $-\left(\lambda_{1}+\lambda_{2}+\cdots+\lambda_{v-1}\right)$.

For each clone, letdenote the vector of the$n$normalization– corrected log-ratio’s obtained from all of the corresponding spots on slides in the experiment. The ordered set of the independent parameters is given by the parameter vector. Therefore, the data can be expressed as a linear model

,,

whereis the vector of independent errors,$I$is the $n\times n$ identity matrix,is the $n\times v$ design matrix describing how the samples are paired onto slides. Each row of$X$corresponds to a spot. For instance, to a spot on the slide in which samples $k$($\neq v$, labeled with Cy5) and $k'$($\neq v$, labeled with Cy3) are mixed and hybridized, the corresponding row is

.

To a spot on the slide in which sample$k$($\neq v$, labeled with Cy5/ Cy3) and sample$v$ (labeled with Cy3/ Cy5) are mixed and hybridized, the corresponding row is of the following form:

For the experiment shown in Figure 1, the equation is as following:


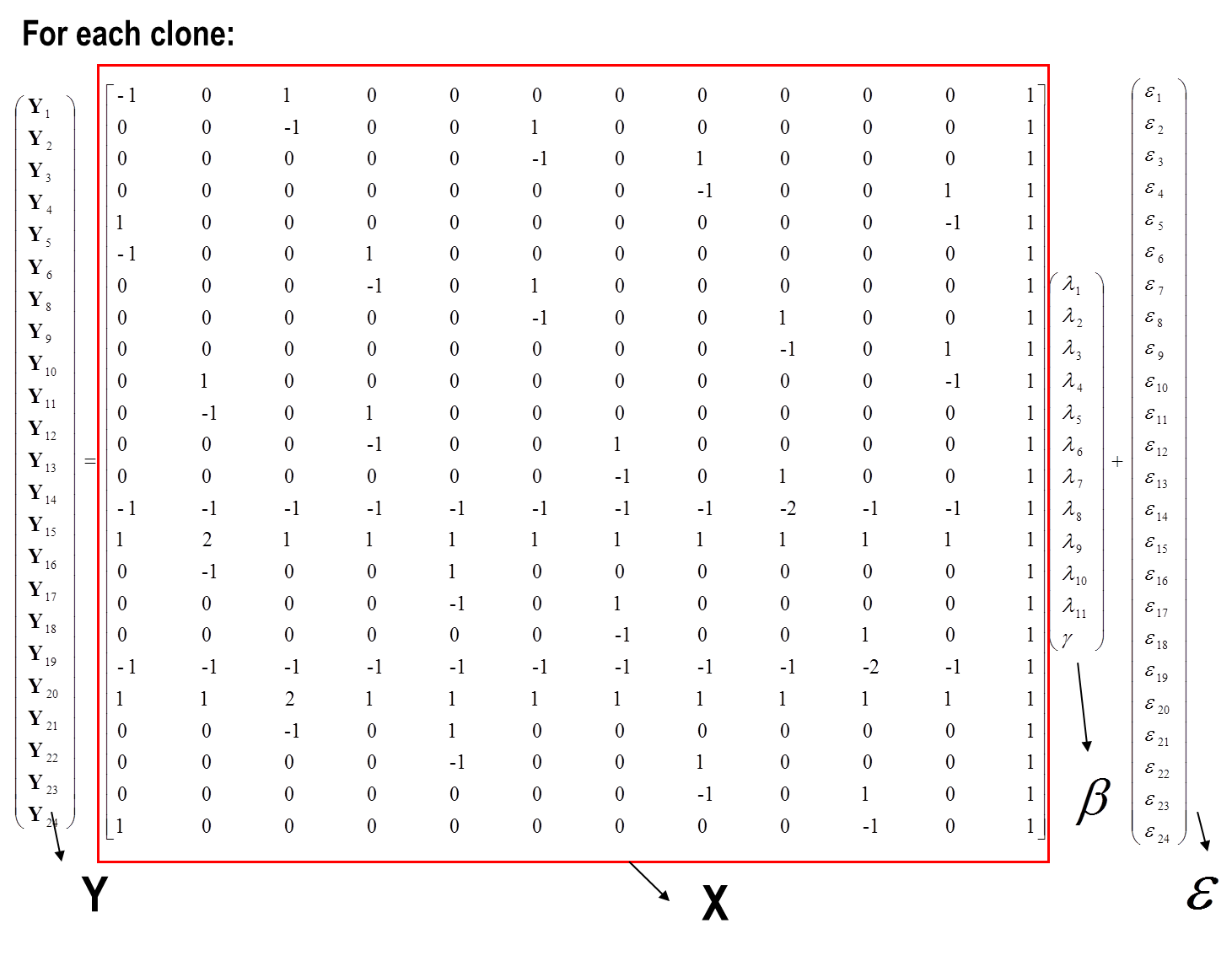


Here we assume the situation in which there is a single replicate for each clone on each slide. If the number of replicates for each clone on slides is greater than one, the corresponding row should be repeated in the matrix, **X**, accordingly.

For each clone, the parameters $\beta$ and $\varepsilon$ is estimated by $\hat{\beta}=(X'X)^{-1}\cdot X'\cdot\underline{Y}$ and $\hat{\varepsilon}=\text{Y-X}\hat{\beta}$. Therefore, .

**Permutation Method**

Differential expression is is denoted as , where *x* is the index denoting clones and *i,j* denoting samples. Differential expression profiles in Figure 2a are the histograms of data set S1:, S2:, and S3:, which are the set of all $d(i,j,x)$ when *x* runs over all clones and (*i,j*) runs over all possible pairs in G1, G2, and G3, respectively. For S1, *i* and *j* range from 1 to 9. For S2, i and j range from 8-1 to 8-3. For S3, i and j are 8-3_1 and 8-3_2 respectively.

In the following, we use D1 as example for permutation. There are

5,501(number of expressed clones) x $C_{2}^{9}$(number of all possible pairs)

=5,501 x 36

=198,036 data in S1.

D1 is the result of ten million times of permutation of (random sampling n data of S1 for one time), where n is the number of sample pair i,j in the experiment and we use it to describe the individual variance in D quantity.
